# The β‐domain of streptokinase affects several functionalities, including specific/proteolytic activity kinetics

**DOI:** 10.1002/2211-5463.12657

**Published:** 2019-05-30

**Authors:** Maryam Rafipour, Malihe Keramati, Mohammad Mehdi Aslani, Arash Arashkia, Farzin Roohvand

**Affiliations:** ^1^ Virology Department Pasteur Institute of Iran Tehran Iran; ^2^ Microbiology Department Pasteur Institute of Iran Tehran Iran; ^3^ NanoBiotechnology Department Pasteur Institute of Iran Tehran Iran

**Keywords:** fibrinolysis, plasminogen activation, protein domains, streptococci clusters, streptokinase, α2‐antiplasmin resistance

## Abstract

Streptokinase (SK) is a plasminogen activator which converts inactive plasminogen (Pg) to active plasmin (Pm), which cleaves fibrin clots. SK secreted by groups A, C, and G *Streptococcus* (SKA/SKC/SKG) is composed of three domains: SKα, SKβ and SKγ. Previous domain‐swapping studies between SK1/SK2b‐cluster variants revealed that SKβ plays a major role in the activation of human Pg. Here, we carried out domain‐swapping between *skcg*‐SK/SK2‐cluster variants to determine the involvement of SKβ in several SK functionalities, including specific/proteolytic activity kinetics, fibrinogen‐bound Pg activation and α_2_‐antiplasmin resistance. Our results indicate that SKβ has a minor to determining role in these diverse functionalities for *skcg*‐SK and SK2b variants, which might potentially be accompanied by few critical residues acting as hot spots. Our findings enhance our understanding of the roles of SKβ and hot spots in different functional characteristics of SK clusters and may aid in the engineering of fibrin‐specific variants of SK for breaking down blood clots with potentially higher efficacy and safety.

**Abbreviations**
Fg: fibrinogen
PA: plasminogen activator
PAM: plasminogen‐binding group A streptococcal M‐like protein
Pg: plasminogen
Pm: plasmin
SA*: specific activity
SK: streptokinase
SKA/SKC/SKG: respectively, groups A, C and G *Streptococcus*
α_2_‐AP: alpha 2‐antiplasmin

Streptokinase (SK), a plasminogen activator (PA) secreted by groups A, C and G streptococci (GAS, GCS and GGS, respectively), converts the inactive plasminogen (Pg) to the active plasmin (Pm) which cleaves the fibrin clots. Despite being considered as a virulence factor (especially in GAS pathogenesis), traditionally, a nonfibrin‐specific SK, isolated from the less virulent GCS (H46A or ATCC9542), was widely used as a fibrinolytic drug 1, 2. PA activity of SK is accomplished in two pathways. First, it binds to Pg and forms a 1 : 1 binary SK‐Pg* activator (amidolytic) complex, which converts the free Pg substrate to Pm (nonfibrin‐specific pathway I). Subsequently, the generated Pm binds SK to form SK‐Pm proteolytic activator complex which converts Pg molecules to Pm (fibrin‐specific pathway II) 2, 3.

The 414 amino acid, SK, is composed of three distinct structural domains: α, β and γ spanning residues: 1–146, 147–290 and 291–414, respectively*.* Protein engineering studies indicated the importance of all three domains and the potential role of several critical amino acids (hot spots), such as Ile1 3, Lys256, Lys257 4 and recently Ile33, Asn228 and Phe287 5, for SK functionality. The attained information was used to improve the fibrinolytic characteristics of SK for enhanced PA potency, fibrin specificity and resistance to the inhibitory effect of plasma α_2_‐antiplasmin (α_2_‐AP). Concurrently, heterogeneity of SKs at the gene (*sk*) and protein (SK) levels in different strains (even the same group) of streptococci (specifically for GAS) and its relation to functional differences was shown 2. Studies indicated the highest sequence diversity of β‐domain compared to α and γ, particularly in a distinct hypervariable region (*sk*‐V_1_; residues 147–218). Accordingly, the *sk*‐V_1_ was suggested as the main source of *sk* allelic variations, and consequently, phylogenetic analysis of the *sk*‐V1 nucleotide sequences was used to classify the GAS‐SK (*ska*) alleles into two main clusters; SK1 and SK2, in which SK2 was further subdivided into subclusters SK2a and SK2b 6, 7. These clusters successfully classified GAS strains into those that contain (a) a Pg/Pm direct binding M‐like protein, ‘PAM’ and usually induce invasive skin infections (SK2b), (b) a fibrinogen (Fg) binding M1 protein that does not directly interact with Pg and usually induce upper respiratory tracts (UTR) infections (SK2a) and (c) a M protein that does not interact with either Pg or Fg (SK1) and optimally activates Pg in solution. Although presence of Fg generally enhances the PA activity of all SK types, the specific characteristic of SK1 for optimal Pg activation in solution is in contrast to the SK2 groups (specially SK2b) which strictly require the presence of Fg to display PA activity 6, 7, 8, 9. Interestingly, GCS/GGS‐SKs (*skcg*), despite expressing Pg‐binding proteins different from PAM and other GAS‐M proteins and displaying high Pg activation in solution (similar to SK1), are clustered in SK2a section of the phylogenetic tree 6. Indeed, most of the *ska* alleles in SK2a cluster are homologous to *skcg* than SK1 or SK2b. Moreover, complexes of Pm with SK2a and *skcg‐*SK display higher resistance to inhibition by α_2_‐AP than SK1 or SK2b 1. Therefore, *skcg*‐SKs display some characteristics specific for either SK1 or SK2a clusters and are thus interesting candidates for comparative studies.

Attempts to address the role of β‐domain heterogeneity for functional characteristics of SK clusters/subclusters started with a study on exchange and swapping the major polymorphic regions between SK1 β‐domain (SK1β) and SK2aβ 10. However, apparently due to the similar PA potencies of the used SK1 and SK2a variants, this study failed to uncover any effect on Pg activation kinetics of the chimeric and parental SKs. Recently, two other studies addressing the domain‐exchange strategies between a SK1 (with high PA activation rate) and SK2b indicated the major role of the β‐domain in the PA activity, which might be further assisted by α‐domain 11. But how β‐domain exchange might alter the kinetics of the amidolytic/proteolytic pathways, Fg‐bound‐Pg activation or the resistance to inhibition by α_2_‐AP, specially between *skcg‐*SK and SK2b, are other concerns that never addressed. Recently, SK from a newly isolated GGS (SKG88) with high PA activity was introduced 12. In the present report, using SKG88 and two other SKs belonging to SK2a and SK2b and employing domain‐exchange approaches, these concerns are addressed.

## Materials and methods

### Bacterial strains and reassessment of the SK clusters

The *skcg*‐SK of GGS (G88) with high PA activities 12, which was supposed to be clustered as SK2a 6, was used for β‐domain exchange between SK of two GAS strains; STAB902 containing SK2a with very low PA activities 13 and ALAB49 (gifted by Mc. Arthur, University of Wollongong, Australia), containing a well‐known SK2b with barely detectable PA activities in culture supernatants 7. The gene accession numbers are as follows: HM390000.1, CP007041.1 and AY234134, respectively. Sequence alignment and phylogenetic analysis of *sk*β‐V1 region for these three SKs and six other well‐known SK clusters 6, 7 were accomplished by ‘Molecular Evolutionary Genetics Analysis, mega6’ 14.

### Construction of the parental and β‐domain‐exchanged SK‐encoding plasmids

The detailed steps for cloning of *sk* into pET26b vector to construct the parental plasmids (pET26b‐SK_G88_, pET26b‐SK_ALAB49_ and pET26b‐SK_STAB902_) are illustrated in Fig. S2.

**Figure 1:**
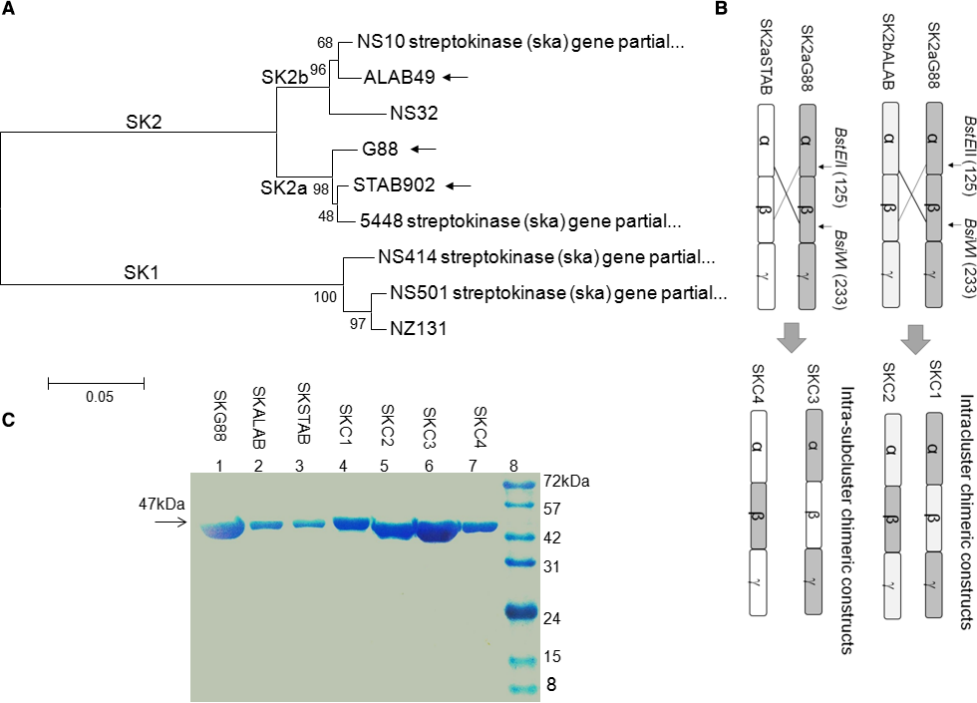
Reassessment of the SK cluster, SKβ‐exchange and analysis of the purified SKs. (A) Phylogenetic analysis on *sk*β‐V1 region for three parental SKs (shown by arrows) and six other well‐known SK clusters (NS10; EU352637.1, NS32; EU352630.1, 5448; CP008776.1, NS411; EU352621.1, NS501; EU352616.1, NZ131; CP000829.1) 6, 7 was accomplished by ‘Molecular Evolutionary Genetics Analysis, mega6’ 14. Bootstrap values of 90% (500 replicates) are indicated. Scale bar = 0.05 substitution per site. (B) Construction of chimeric SKs by β‐domain exchange of residues 125–233 between parental SKs (the unique restriction sites used for sequence exchange are indicated). SK_C1_/SK_C2_ and SK_C3_/SK_C4_ were made by β‐domain exchanges between SK2a_G88_/SK2b_ALAB49_ and SK2a_G88_/SK2a_STAB902_, respectively. (C) SDS/PAGE analysis of purified chimeric and parental SK proteins. Lanes 1–7: the purified SKs corresponding to each band were indicated above each well. Lane 8: molecular weight marker (SM7012, Cinnagen Co, Tehran, Iran).

For construction of the β‐domain‐exchanged SKs, the region corresponding to nucleotides 375–699 (residues 125–233) from the parental vectors was digested by *BstE*II/*BsiW*I restriction enzymes and the digested fragments (327 bp) were cross ligated between SK_G88_ and two other SKs (SK_ALAB49_ and SK_STAB902_) (Fig. 1B). All the molecular methods were based on the standard protocols 15.

### Expression, purification and characterization of SK proteins

*Escherichia coli* Rosetta (Novagen, USA) was used for protein expression via IPTG induction, and expressed SKs were purified under native conditions using nickel‐nitriloacetic acid (Ni‐NTA) affinity chromatography (Qiagen, USA) according to the manufacturer’s protocols (QIA‐expressionist 2002; Qiagen). Protein concentrations were determined by Bradford assay. Expression and the purity of the purified SKs were assessed by 12% (w/v) SDS/PAGE and confirmed by western blotting. Protein characterizations assays are described in Figs S5 and S6.

### Determination of SK‐specific activity (SA*)

**Figure 2:**
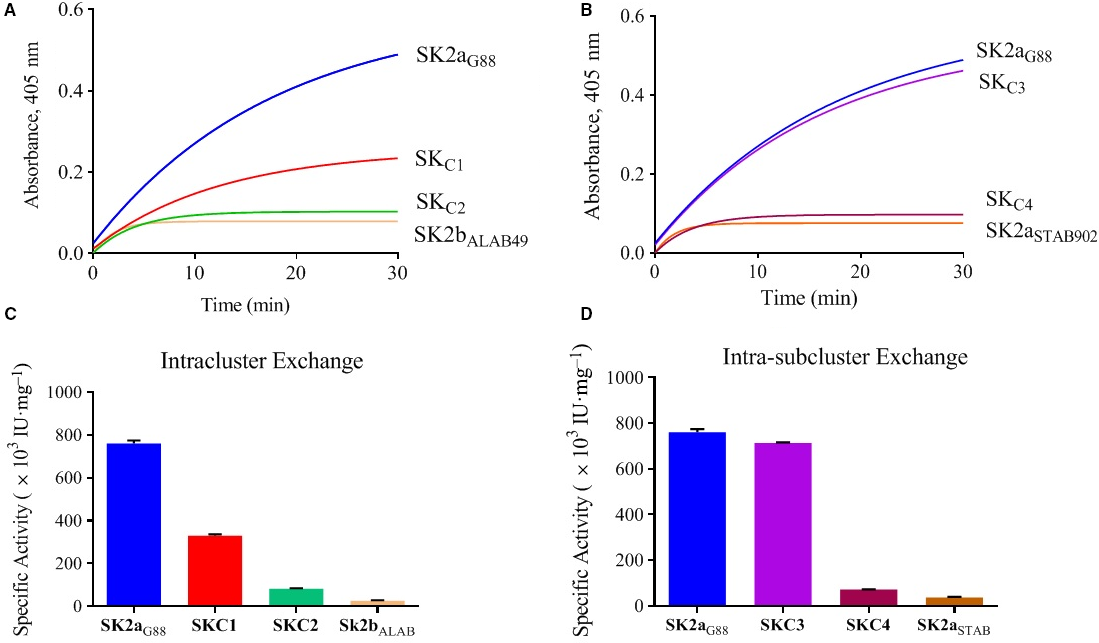
Determination of PA activity of SKs by chromogenic assay using S‐2251 substrate. The standard chromogenic assay by the synthetic chromogenic substrate, ‘tripeptide H‐d‐valyl‐l‐leucyl‐l‐lysine‐*p*‐nitroanilide dihydrochloride (S‐2251; Sigma)’ which is an approved assay for determination of SK activity (Third International Standard for streptokinase; National Institute of Biological Standards and Controls, NIBSC, 2004UK), was used throughout the study. For determination of specific activities (SA*), purified SK proteins (100 nm) were added to a microtitre plate containing 1 mm of S‐2251 and 1 µm of Pg (Sigma) in a total volume of 100 µL of assay buffer (50 mm Tris/HCl, pH 7.4). Hydrolysis of S‐2251 was measured at 405 nm every 5 min for 60 min in a microplate reader (Synergy^TM^ 4, BioTek‐Fisher Scientific, UK). All reactions were performed in triplicate. Serial dilutions of Streptase^®^ (CSL, Behring, Germany), a commercially available standard SK, were used to prepare the standard calibration curve (Fig. S7) which was needed for calculating SK‐specific activities (SA*). For calculation of the SA*, the time‐course activity profiles (the change in absorbance at 405 nm as a function of time) were measured (Fig. 2A,B), and subsequently, SA* (Fig. 2C,D) was calculated from the slope of the linear portion of the curve (Fig. 3) in which serial dilutions of commercial (standard) SK (Streptase) were used as the reference for calibration (Fig. S7). SK2a_G88_, SK2b_ALAB49_ and SK2a_STAB902_ are parental constructs. SK_C1_ (2a_G88_‐2b_ALAB_‐2a_G88_) and SK_C2_ (2b_ALAB_‐2a_G88_‐2b_ALAB_) denote the intracluster chimeras. SK_C3_ (2a_G88_‐2a_STAB_‐2a_G88_) and SK_C4_ (2a_STAB_‐2a_G88_‐2a_STAB_) denote the intra‐subcluster chimeras. Data represent the mean ± SD of triplicate experiments.

**Figure 3:**
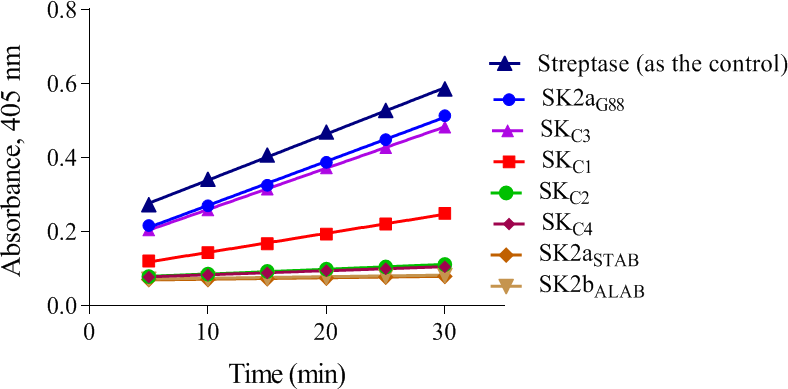
The time‐course activity profiles (the change in absorbance at 405 nm as a function of time) of SKs. For measuring the specific activity (IU·mg^−1^) of the SKs, the slopes of the linear portion of the curve obtained from plotting absorbance (OD) at 405 nm vs time (Fig. 2A,B) were used. Serial dilutions of Streptase^®^ (CSL) were used as reference for preparation of the standard curve (Fig. S7) and calibration of international units·mg^−1^ protein (specific activity) in the samples. SK2a_G88_, SK2b_ALAB49_ and SK2a_STAB902_ are parental constructs. SK_C1_ (2a_G88_‐2b_ALAB_‐2a_G88_) and SK_C2_ (2b_ALAB_‐2a_G88_‐2b_ALAB_) denote the intracluster chimeras. SK_C3_ (2a_G88_‐2a_STAB_‐2a_G88_) and SK_C4_ (2a_STAB_‐2a_G88_‐2a_STAB_) denote the intra‐subcluster chimeras.

For evaluation of the SA* in the presence/absence of Fg, the standard colorimetric assay using the chromogenic substrate (S‐2251; Sigma, USA) was used throughout this study, as previously described 7. The detailed procedure for the assay, construction of the calibration curve and calculation of the SA* are provided in Fig. S7, Figs 2 and 3.

### Determination of kinetic constants for amidolytic and proteolytic activities

For analysing amidolytic kinetics, first stoichiometric concentrations of Pg and SK (5.5 µm SK and 5 µm Pg) were mixed and incubated for 5 min to produce the SK‐Pg* activator complex. Subsequently, an aliquot of the complex (100 nm) was transferred to the assay buffer along with various concentrations of S2251 (0.1–1.5 mm) in a total volume of 100 µL 12.

For analysing proteolytic kinetics, 100 nm of SK was added to assay buffer containing, ‘0.1 mm S2251 and varying concentrations of Pg (0.3–5.0 µm)’ and changes in absorbance at 405 nm were monitored for 30 min. The data were plotted as velocity/substrate concentration, and kinetic parameters of Pg activation were determined from Michaelis–Menten (*V* vs *S*) and inverse (1/*V* vs 1/*S*) Lineweaver–Burk plots using graphpad prism 6 (GraphPad Software, La Jolla, CA, USA) 12.

### Inhibition by α_2_‐antiplasmin

Stoichiometric complexes of SK‐Pm (400 nm SK and 200 nm Pm) were incubated for 5 min. The complex was diluted to 20 nm in assay buffer containing α_2_‐AP (final concentration: 100–400 nm). The mixtures were incubated for 15 min, then S‐2251 (500 µm) was added to the reaction, and residual activity of complex was measured by change in absorbance at 405 nm 1 *.*

### Statistical analyses

Unpaired, two‐tailed Student’s *t* test with 95% confidence intervals was used for analysis of SK‐PA activities and kinetics using spss software version 22.0 (SPSS Inc., USA). All linear regressions were by graphpad prism 6, and *P*‐values < 0.05 were considered significant.

## Results and Discussion

### Confirmation of the SK clusters and production of SK proteins

Sequence alignment of *sk*β‐V1 region for G88, STAB902 and ALAB49 and six other well‐known SK clusters 6, 7 that were used for construction of the phylogenetic tree is provided in Fig. S1. Phylogenetic analysis (Fig. 1A) identified the *skg*‐encoded‐SKG88 as SK2a (SK2a_G88_) which is in complete agreement with prior reports on clustering of *skcg* alleles 6. Accordingly, STAB902 and ALAB49 SKs were subclustered as SK2a (SK2a_STAB902_) and SK2b (SK2b_ALAB49_), respectively 7, 13. PCR amplification of all three parental SKs produced the expected 1250‐bp amplicon (Fig. S3). Cloning steps for insertion of parental SKs (SK2a_G88_, SK2b_ALAB49_, SK2a_STAB902_) into pET26b vector and exchange of the 327‐bp β‐domain (*BstE*II/*BsiW*I) fragments between SK2a_G88_ and SK2b_ALAB49_ (hereafter intracluster chimeric constructs; SK_C1_ and SK_C2_) and between SK2a_G88_ and SK2a_STAB902_ (hereafter intra‐subcluster chimeric constructs; SK_C3_ and SK_C4_) vectors are illustrated in Fig. S2 and Fig. 1B, respectively. Restriction analyses of the recombinant SK‐encoding vectors (Fig. S4) and nucleotide sequence analysis (Fig. S8) confirmed the accuracy of the cloning procedures. Characterization of the expressed SK proteins by SDS/PAGE (Fig. S5A) and western blotting (Fig. S6), in accordance with the prior reports 11, 12, 16, indicated the expression of SKs with the expected size (47 kDa). Purification by one‐step Ni‐NTA affinity chromatography resulted in 90% purity (Fig. 1C and Fig. S5B).

### Contribution of the SKβ in specific activity (SA*)

For calculation of the SA*, the time‐course activity profiles (the change in absorbance at 405 nm as a function of time) were measured (Fig. 2A,B), and subsequently, SA* was calculated (Fig. 2C,D and Table 1), from the slope of the linear portion of the curve (Fig. 3) in which serial dilutions of commercial/standard SK (Streptase) were used as the reference for calibration (Fig. S7). As shown in Table 1, the SA* of SK2a_G88_ (760.82 × 10^3^ IU·mg^−1^) was about 28‐fold and 22‐fold higher than that of SK2b_ALAB49_ (26.64 × 10^3^ IU·mg^−1^) and SK2a_STAB902_ (36.50 × 10^3^ IU·mg^−1^), respectively. Prior studies reported over 10‐fold higher PA activity for SK1 compared to SK2b 8, 11 which further supports the similarity of SK1 and *skcg*‐SK (SK2a_G88_) for optimal PA activity in solution 11. Indeed, SK1β is the most divergent among all SK clusters, and the divergence between SK1 and SK2b might even exceed 40% 6, 7, which might further support the determining role of β‐domain for functional characteristics between SK1 and SK2b clusters 11, 16. But sequence alignments (Fig. S8 and Table 3) indicated that the exchanged β‐domains between SK2a_G88 _and SK2b_ALAB49_ (intracluster; Fig. 1B) were 89% similar, while α‐ and γ‐domains exhibited 82% and 86% similarity, respectively. Therefore α‐ and γ‐domains might have more contribution in functional characteristics of the *skcg*/SK2b domain‐exchanged SKs in our study (SK_C1_/SK_C2_) than that of SK1/SK2b in the prior report 11. In contrast, exchanging the SK2a_G88_β and SK2a_STAB902_β, for making the two intra‐subcluster constructs (SK_C3_; 2a_G88_‐2a_STAB_‐2a_G88_ and SK_C4_; 2a_STAB_‐2a_G88_‐2a_STAB_; Fig. 1B) led to less alterations in the SA* values for SK_C3_/SK_C4_ compared to SK2a_G88_ and SK2b_ALAB49_ (Fig. 2D and Table 1). Thus, our results, consistent with a prior study on SK1β and SK2aβ exchanged domains, could not uncover any major effects on PA potencies 10. However, in the prior study, despite sharing less than 50% identity between exchanged SK1β and SK2aβ, the parental SKs had relatively similar SA* 10, while despite clustering as SK2a, the SK2a_G88_ and SK2a_STAB902_ in our study show highly different SA* (Table 1). Indeed, the exchanged β‐domains (residues 128–233) of SK2a_G88_ and SK2a_STAB902_ were around 97% identical (corresponding to only three residue substitutions out of 108; K138S, I151V, E161K; Table 3) while their α‐ and γ‐domains exhibited 85% and 88% similarity, respectively (Fig. S8). Therefore, it might be the presence of only few scattered residues acting as hot spots rather than accumulated altered residues in a specific domain that counts for the highly different SA* activities, as recently claimed 5. Having shown that β‐domain exchange between SK2a intra‐subclusters (SK_C3_/SK_C4_) had little contribution to SA*, the rest of the experiments were only performed for SK_C1_/SK_C2_.

**Table 1:** Specific activities of SK variants.

Parental SK^a^	Specific activity (× 10^3^ IU·mg^−1^)	Chimeric SK^a^		Specific activity (× 10^3^ IU·mg^−1^)
SK2a_G88_ (α2aβ2aγ2a)	760.82 ± 13.63	Intracluster	SK_C1 _(2a_G88_‐2b_ALAB_‐2a_G88_)	329.24 ± 7.07
			SK_C2 _(2b_ALAB_‐2a_G88_‐2b_ALAB_)	83.24 ± 1.28
SK2b_ALAB49 _(α2bβ2bγ2b)	26.64 ± 1.79			
		Intra‐subcluster	SK_C3 _(2a_G88_‐2a_STAB_‐2a_G88_)	713.54 ± 2.64
SK2a_STAB902 _(α2aβ2aγ2a)	36.50 ± 1.02			
			SK_C4 _(2a_STAB_‐2a_G88_‐2a_STAB_)	70.84 ± 0.89

All measured *P*‐values were less than 0.05 (*P* < 0.05) considered significant.

a Parental and chimeric SK denote the three originally isolated SKs and four SKβ‐exchanged SKs. Please see the text for complete explanations.

### Contribution of the SKβ in the kinetics of amidolytic/proteolytic activity

Amidolytic/proteolytic activity of the SKs was studied by measuring the steady‐state kinetic constants of the S2251 hydrolysis including substrate affinity (*K*_m_), catalytic activity (*K*_cat_) and the constant of catalytic efficiency (*K*_cat_/*K*_m_; efficiency of the Pg conversion into Pm). As shown in Table 2, *K*_m_ and *K*_cat_ values did not alter significantly between SK_C1_ and SK2a_G88_ (0.39 mm and 1.39 s^−1^ vs 0.41 mm and 1.39 s^−1^, respectively) leading to almost similar catalytic efficiency (*K*_cat_/*K*_m_). For SK_C2_ compared to SK2b_ALAB49_, the *K*_cat_ raised by 2.5% (0.88 vs 0.86 s^−1^) and the *K*_m_ reduced by 17% (0.34 vs 0.41 mm) leading to an overall 24% increase in catalytic efficiency (2.59 × 10^3^ vs 2.10 × 10^3^ s^−1^·m ^−1^) (Table 2). Interestingly, evaluation of the kinetic parameters for proteolytic activity indicated that the catalytic efficiency of SK_C1_ declined by 47% compared to SK2a_G88_ (229.27 × 10^3^ vs 428.57 × 10^3^ s^−1^·m ^−1^), which was mainly due to threefold increase in *K*_m_ value (0.77 vs 2.05 µm). For SK_C2_ compared to SK2b_ALAB49_, the *K*_m_ declined by 57% (3.45 vs 7.92 µm) and the *K*_cat_ increased by 40% (0.22 vs 0.16 s^−1^) leading to more than threefold augmented values for catalytic efficiency (63.77 × 10^3^ vs 20.20 × 10^3^ s^−1^·m ^−1^). These results indicated the determining role of the *skcg*β (SK2a_G88_β) on enhancement of proteolytic activity (Table 2), mainly due to the augmentation of the *K*_m_ values (increased substrate affinity) which is in accordance with the SA* results (Table 1). Our results are consistent with a prior report on the importance of the SKβ for strong binding of Pg substrate to the SK‐Pm proteolytic complex and its efficient conversion to Pm 17.

**Table 2:** Kinetic parameters for amidolytic/proteolytic activities of SK variants.

SK variants	Amidolytic constants			Proteolytic constants		
	*K*_m_ × 10^−3 ^(m)	*K*_cat_ (s^−1^)	*K*_cat_/*K*_m_ × 10^3^ (s^−1^·m ^−1^)	*K*_m_ × 10^−6^ (m)	*K*_cat_ (s^−1^)	*K*_cat_/*K*_m_ × 10^3^ (s^−1^·m ^−1^)
SK2a_G88_	0.41 ± 0.006	1.39 ± 0.031	3.39	0.77 ± 0.14	0.33 ± 0.082	428.57
SK2b_ALAB49_	0.41 ± 0.048	0.86 ± 0.122	2.10	7.92 ± 0.16	0.16 ± 0.004	20.20
SK_C1_	0.39 ± 0.062	1.39 ± 0.092	3.56	2.05 ± 0.753	0.47 ± 0.237	229.27
SK_C2_	0.34 ± 0.052	0.88 ± 0.090	2.59	3.45 ± 0.23	0.22 ± 0.018	63.77

All measured *P*‐values were less than 0.05 (*P* < 0.05) considered significant.

### Contribution of the SKβ on Fg‐bound‐Pg activation

**Figure 4:**
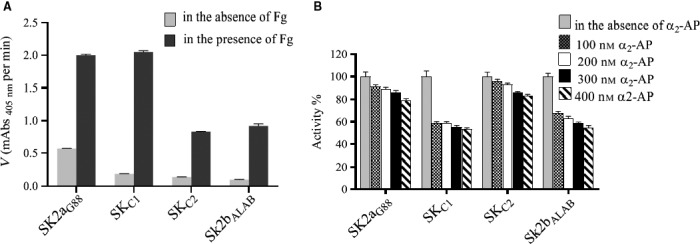
Comparison the activity of SK variants in the absence/presence of (A) fibrinogen (Fg) or (B) α_2_‐antiplasmin (α_2_‐AP) (A) Measurement of SK activity in the presence of Fg was also based on hydrolysis of S‐2251 by Pg. Fg (1 µm) and Pg (1 µm) were mixed (1 : 1 stoichiometric ratio) and pre‐incubated for 15 min. Subsequently, SK (100 nm) and S‐2251 (1 mm) were added to the mixture. The hPg activation rate of various SKs in presence and absence of Fg was measured by monitoring the absorbance at 450 nm and calculated by linear regression from the linear regions of plots A405 nm vs time (Fig. 5). The activation rate of all constructs belonging to either SK2a or SK2b cluster improved several orders of magnitude ranging from 3.5‐fold in case of SK2a_G88 _to 10.8‐fold in case of SK_C1 _in the presence of hFg. Notably, Fg stimulates the activity of cluster 2b more efficiently than that of cluster 2a. (B) The inhibitory effect of α_2_‐AP on SK‐hPn complex activity in different concentrations of the inhibitor was illustrated relative (%) to the activity of the complex in absence of the inhibitor. Stoichiometric complexes of SK‐hPn (20 nm) were performed for 5 min at 37 °C and incubated for 15 min at 37 °C with α_2_‐AP (final concentration 100–400 nm). The reactions were initiated by addition of S‐2251 (500 mm) to complex mixtures, and the activity of the SK‐hPn complexes was determined by measuring the absorbance at 405 nm. All SK‐hPn complexes resisted the inhibitory effect of α_2_‐AP, but to different degrees, SK2a retained more than 80% of the activity in presence of the maximum concentration of α_2_‐AP, whereas SK2b residual activity in the highest concentrations of the inhibitor was almost 50%. SK_C1 _(2a_G88_‐2b_ALAB_‐2a_G88_) and SK_C2_ (2b_ALAB_‐2a_G88_‐2b_ALAB_) are the intracluster β‐domain‐exchanged variants; SK2a_G88_ and SK2b_ALAB49_ are the parental SKs. Data represent the mean ± SD of triplicate experiments. All measured *P*‐values were less than 0.05 (*P* < 0.05) considered significant.

**Figure 5:**
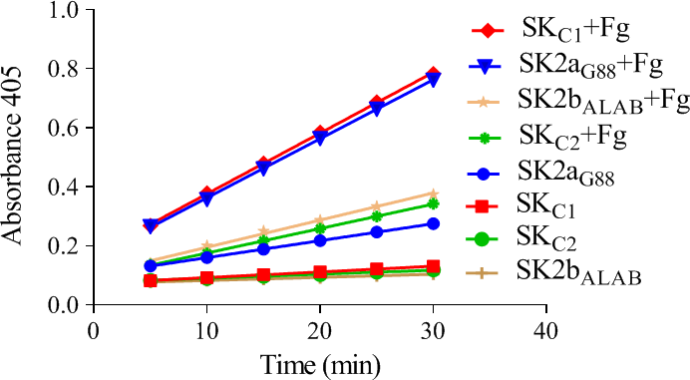
The time‐course activity profiles (the change in absorbance at 405 nm as a function of time) of SKs, in the presence/absence of fibrinogen (Fg). The Pg activation rates were measured by monitoring the absorbance at 450 nm and calculated by linear regression from the linear regions of plots A405 nm vs time. In the presence of Fg, activation rate (ΔOD405/t) of all SKs raised significantly but the effect is more reflective for SK2bβ containing constructs (SK_C1 _and SK2b_ALAB49_). SK2aG88 and SK2b_ALAB49_ are parental constructs. SK_C1_ (2a_G88_‐2b_ALAB_‐2a_G88_) and SK_C2_ (2b_ALAB_‐2a_G88_‐2b_ALAB_) denote the intracluster chimeras.

The Pg activation rate of various SKs in the presence/absence of Fg was measured by monitoring the absorbance at 450 nm and calculated by linear regression from the linear regions of the plots (OD_405_/time). As shown in Figs 4A and 5, generally, in the presence of Fg, activation rate (ΔOD_405_/t) of all SKs (SK2a_G88,_ SK_C1_, SK_C2_, SK2b_ALAB49_) raised significantly (2.00, 2.00, 0.83, 0.92 × 10^−2^ vs 0.57, 0.19, 0.14, 0.10 × 10^−2^, respectively), but the effect is more reflective for SK2bβ containing constructs (SK_C1/_SK2b_ALAB49_) than SK2a_G88_/SK_C2_ (10.80/9.20 vs 3.51/5.93‐fold enhancement of activation rates, respectively)._._These results are consistent with prior reports for higher influence of Fg on enhancement of SK2b activity compared to SK1 7, 8 and that of the SK2a compared to a *skc*‐SK 9, indicating more similarity of the *skcg* (SK2a_G88_) to SK1 variants for activation of Fg‐bound‐Pm. As shown in Fig. 4A and consistent with a recent study 12, SK2a_G88_ showed high intrinsic Fg‐bound‐Pg activation. This higher Fg‐bound‐Pg activation (twofold higher than SK2b_ALAB49_; 2.00 vs 0.92) is completely retained in SK_C1,_ while in the absence of Fg, PA potency of SK_C1_ is three times lower than the parental SK2a_G88_ (0.19 vs 0.57). Of note, these characteristics of SK_C1_ might be of interest for development of a fibrin‐specific version of SK for targeted fibrinolysis 3. Interestingly, SK_C2 _retained the same (and low) activity as SK2b_ALAB49_ in the absence of Fg, while its Fg‐bound‐Pg activation showed 42% (0.83 vs 2.00) and 90% (0.83 vs 0.92) of the parental activity (SK2a_G88_ and SK2b_ALAB49_, respectively). Collectively, while these results support the major contribution of the SKβ for the Fg‐bound‐Pg, but in agreement with prior reports also implies the potential contribution of other domains for this characteristic 3.

### Contribution of the SKβ on resistance to α_2_‐AP inhibition

As shown in Fig. 4B, all four SK‐Pm complexes resisted the inhibitory effect of α_2_‐AP (400 nm) by retaining more than 50% of their activity. This observation is consistent with the long‐known phenomena for resistance of the SK‐Pm complex to the major physiological plasmin inhibitor ‘α_2_‐AP’ 18. However, for SK2a_G88_ and SK_C2_ (containing SK2a_G88_β), retained activity was about 80%, while for that of SK2b_ALAB49_ and SK_C1_ (containing SK2b_ALAB49._β), it was about 50% (Fig. 4B). Thus, our results indicated that *skcg*‐SK was more resistant to inhibition by α_2_‐AP than SK2b, which is in agreement with recent findings for Pm‐complexed with either SK‐H46A (*skc*) or SK2a variants 1. Therefore, our results indicated the resemblance of *skcg‐*SK to SK2a variants for ‘α_2_‐AP resistance’ and the determining role of SKβ in these characteristic. Although this finding is consistent with prior suggestion on the contribution of SKβ to the interaction of SK with inhibitors 19, the role of other domains, specially residue 1–59 of α‐domain for resistance to α_2_‐AP, was also suggested 3.

### Potential contribution of the substituted residues (hot spots) in SK functionalities

As emphasized earlier, a recent study on a new isolate of *skcg*‐SK (GGS‐132) indicated that presence of only three altered residues (Ile33Phe, Asn228Lys and Phe287Ile) that probably acted in a synergic mode as hot spots might induce enhanced proteolytic/Fg‐bound‐Pg activation compared to a SKC (GCS‐SKC9542) 5, 12. Accordingly, it was also shown that SK2a_G88,_ in the present study, exhibited enhanced proteolytic/Fg‐bound‐Pg activation compared to the same SKC, while only seven residues scattered within domains of the two SKs were substituted (98% similarity) 12. As shown in Table 3, the exchanged segments between SK2a_G88_β and SK2b_ALAB49_β differ by 12 residues. Among these altered residues, ‘V160I, E161R and K209E’, consistent with a recent report 17, might have potentially acted as hot spots for the induced functionalities of the domain‐swapped SKs (SK_C1_/SK_C2_). The precise insight on the effect of these substitutions might be gained via site‐directed mutagenesis experiments.

**Table 3:** The altered residues in the exchanged SKβ domain of SK2a_G88_ compared to SK2a_STAB902_ and SK2b_ALAB49_. Conserved (identical) residues are indicated by dots.

SK variant	Residue position												
	132	134	138	151	153	154	160	161	176	178	209	210	213
SK2a_G88_	V	E	K	I	N	Q	V	E	R	G	K	T	G
SK2a_STAB902_	*.*	*.*	S	V	*.*	*.*	*.*	K	*.*	*.*	*.*	*.*	*.*
SK2b_ALAB49_	I	Q	R	*.*	T	P	I	R	K	V	E	S	D

In conclusion, to the best of our knowledge, we reported the first domain‐exchange study for *skcg* and cluster 2‐*ska* alleles to elucidate the contribution of SKβ for a broad range of functional characteristic including kinetics of specific/ proteolytic activity, fibrinogen‐bound Pg activation and α_2_‐antiplasmin resistance. Results pointed to the ‘minor to determining’ contribution of SKβ in these functionalities which might be potentially accompanied by a few critical residues acting as hot spots. Our findings indicated the (a) similarity of coclustered, *skcg*β and SK2aβ variants (only three residues alteration) and minor contribution of their SKβ for highly different SA* between these two alleles; (b) similarity of *skcg to* cluster 1‐*ska* alleles (SK1) for optimal PA activity in solution and activation of Fg‐bound‐Pm compared to that of the SK2 variants and major contribution of SKβ in this characteristic; (c) major role of the SKβ on enhancement of proteolytic activity between *skcg*‐SK and SK2b that is mainly due to the augmentation of the *K*_m_ values (increased substrate affinity);and (d) similarity of *skcg‐*SK *to* SK2a variants for ‘α_2_‐AP resistance’ and the determining role of SKβ in this characteristics. These findings might assist in better understanding of the roles displayed by SKβ and hot spots for different functional characteristic of SK clusters and engineering fibrin‐specific versions of SK.

## Conflict of interest

The authors declare no conflict of interest.

## Author contributions

MR, the Ph.D candidate, did most of the experiments and prepared the primary draft of the manuscript. MK, the 1st advisor, assisted in cloning and kinetics analysis and design of the study. MMA, the 2nd supervisor, assisted in strain isolation and microbiology assays, and design of the study. AA, the 2nd advisor, assisted in protein expression and purification assays. FR, the 1st supervisor, designed and supervised the study and prepared the final manuscript for submission.

## Supporting information


**Fig. S1** **.** Multiple DNA Sequence alignment of *skβ‐*V1 fragments. Multiple Sequence alignment of skβ‐V1 fragments (hypervariable region1 of the β‐domain)from nucleotide “445 to 655” of *sk* gene, corresponding to the amino acid residues “147 to 218” of the SK of the strains used in this study (G88, STAB902 and ALAB49; Genbank Accession numbers: HM390000.1, CP007041.1 and AY234134, respectively) together with the well‐known strains that were employed for construction of phylogenetic tree in Fig. 1A (Genbank Accession numbers: NS10; EU352637.1, NS32; EU352630.1, 5448; CP008776.1, NS414; EU352621.1, NS501; EU352616.1, NZ131; CP000829.1). The alignment was created using MEGA software [14]. Conserved (identical) nucleotides are indicated by dots.
**Fig. S2** **.** Schematic illustration for construction of the parental SK‐encoding plasmids. For construction of the three parental constructs, the genomic DNA from culture of the three targeted streptococci (G88, STAB902 and ALAB49) grown in BHI (brain heart infusion) broth, were isolated by DNA extraction kit (Qiagen, USA) according to the manufacturer’s protocols. The extracted genomic DNA was used as template for PCR‐based amplification of the coding region of *sk* genes (lacking the signal peptide sequence) using a pair of primers with inserted restriction sites (*Nde*I‐* Xho*I) for direct cloning into pET26b vector (forward primer; *Nde*I‐SKf: 5׳‐GACGAGACATATGATTGCTGGACCTGAGTG‐3׳; reverse primer; *Xho*I‐SKr 5׳‐GACACTCGAGTTTGTCGTTAGGGTTATCAG‐3׳, the sequences corresponding to restriction sites are underlined). The resulting amplified fragments were digested with *Nde*I and *Xho*I and cloned into the same sites of pET26b expression vector downstream of T7 promoter, in tandem with the fused C‐terminus 6XHis‐tag to provide the three parental SK‐encoding vectors (pET26b‐SK_G88_, pET26b‐SK_ALAB49_ and pET26b‐SK_STAB902_). All cloning steps were performed according to standard procedures [15]. ATG stands for vector‐derived, translation‐start codon; MCS, multiple cloning sites; 6His‐tag is the tag derived from the vector.
**Fig. S3** **. **Analysis of the PCR‐amplified *sk* genes by agarose gel (1%) electrophoresis. The coding region of *sk* gene (lacking the signal peptide sequence) was amplified by PCR using SKf and SKr primers. PCR reactions resulted in a single band of the expected length (1250bp) of *sk* genes. Lane1: DNA Marker 1kb (Thermo scientific SM0311), Lane2 and 3: PCR products of *skg88* and *skstab902 *gene from genomic DNA. The corresponding bands were indicated by arrows. The sizes of the bands of DNA marker are illustrated on the right.
**Fig. S4** **. **Agarose gel (1%) electrophoresis of the restriction enzyme analysis of the recombinant vector pET26b‐SKG_88_. Lane 1: Digested pET26b‐SKG_88 _by *Bst*EII, produced two bands with the approximate size of 1400 and 5100 bp. Lane 2: Digested pET26b‐SKG_88 _by *Nde*I*‐Xho*I, yielded 5230 and 1250 bp fragments corresponding to vector and PCR fragments, respectively. The corresponding bands were indicated by arrows. Lane 3: DNA Marker 1kb (Thermo scientific SM0311). The same analysis for the other two parental constructs pET26b‐SK_ALAB49_ and pET26b‐SK_STAB902 _produced the same results (not shown).
**Fig. S5** **. **Analysis of the protein expression and purification by SDS/PAGE (12%). For protein expression, first the transformation of *E. coli* Rosetta cells (Novagen, USA) with the seven SK‐encoding recombinant vectors (three parental vectors: pET26b‐SK_G88_, pET26b‐SK_ALAB49_ and pET26b‐SK_STAB902_ and four domain‐exchanged chimeric vectors: pET26b‐SK_C1_ to pET26b‐SK_C4_) by standard CaCl_2_ method was performed. Subsequently, protein expression was induced at OD_600_ of 0.5–0.6 by isopropyl‐β‐D‐thio‐galactoside (IPTG) to a final concentration of 1 mM for 3 hours at 37°C. Finally, cells were harvested by centrifugation and stored at ‐20°C for purification steps. Purification of His‐tagged SK proteins from induced *E.coli Rosetta* cells was performed under native conditions, using nickel‐nitriloacetic acid (Ni‐NTA) affinity chromatography and according to manufacturer’s protocol (QIAexpressionist, 2002, Qiagen company website). Briefly, the cell pellets were resuspended in binding buffer (50 mM NaH_2_PO_4_, 300 mM NaCl, 10 mM imidazole) with 0.5 mg/ml lysozyme at 2–5 ml per gram wet weight. After incubation on ice for 30 min, the cells were disrupted by sonication, and supernatant was collected after centrifugation at 10,000 g for 20‐30 min at 4°C. After addition of 1ml resin Ni‐NTA to the clear lysate, the mixture was shaken at 4°C for 60 minutes, loaded on column and washed 4 times with 4 ml wash buffer (50 mM NaH_2_PO_4_, 300 mM NaCl, 20 mM imidazole) then 4 times with 0.5 ml elution buffer (50 mM NaH_2_PO_4_, 300 mM NaCl, 250 mM imidazole) (QIAexpressionist 2002). SDS‐PAGE analyses were performed according to standard procedures [15]. (A) Analysis of the bacterial crude extracts for expression by pET26b‐SK_G88_. Lanes 1‐3; un‐induced bacterial cells, Lane 4; protein marker 10‐180 kDa (SM7012 CinnaGen Co), Lanes 5‐8; corresponds to IPTG induced cells with 0.3, 0.5, 0.7, 1 and 1.2 mM IPTG, respectively. (B) Analysis of protein purification steps for pET26b‐SK_G88_. Lanes 1‐ 4 represent the elution E1 to E4, respectively collected after protein extraction under native condition. Lane5: protein marker 10‐180 kDa (SM7012 CinnaGen Co). Arrows indicate the location of 47 kDa (SK). Analysis of other constructs for expression and purification produced the same results (not shown).
**Fig. S6** **. **Confirmation of the expressed proteins by western blotting. Western blotting was performed according to the standard protocols [15]. Briefly, proteins were transferred from SDS‐PAG to the nitrocellulose membrane and the membrane was blocked by 5% BSA. Mouse anti‐His monoclonal antibody (Qiagen, USA) was used as the primary antibody and goat anti‐mouse IgG conjugated to HRP (Horse Radish peroxidase) (Qiagen, USA) as the secondary (tracking) antibody. Detection of the bands was by 3, 3‐ diaminobenzidine (DAB) (Qiagen, USA). Western‐blot analysis for SK2a_G88_ and SK_C1_ proteins are shown. Lane 1; molecular weight marker (SM7012, Cinnagen Co), lanes 2 and 4; crude lysis of *E. coli* Rosetta cells after induction by IPTG (1mM), expressing SK2a_G88_ and SK_C1_, respectively, lane 3; crude lysis of *E. coli* Rosetta cells before induction (no band was observed). Lanes are spliced together to remove an intervening lane and the vertical dotted line is at the location of the spliced lanes. The arrow indicates the location of 47 kDa (SK).
**Fig. S7** **. **Calibration curve for standard SK. Serial dilutions of Streptase® (CSL, Behring, Germany), a commercially available standard SK, were used to prepare the standard calibration curve based on Hydrolysis of S‐2251 by Pg, as explained in Fig. 2 and Fig. 3.
**Fig. S8** **. **Amino acid sequence alignment of SK proteins corresponding to reference strain SK_9542 _(*S. equisimilis*, ATCC9542, the commercial source for production of SK), SK2b_ALAB49_, SK2a_G88_, and SK2a_STAB902_. The alignment was created using MEGA6 software [14]. Conserved (identical) amino acids in the alignment are indicated by dots. The exchanged fragments are highlighted.
