# Supplementary material for: The β‐domain of streptokinase affects several functionalities, including specific/proteolytic activity kinetics
Source: FEBS Open Bio. 2019 May 30;9(7):1259–69. doi: 10.1002/2211-5463.12657 (PMC6609762; doi:10.1002/2211-5463.12657)
Supplement: Supplementary file 1 — Fig. S1 . Multiple DNA Sequence alignment of skβ‐V1 fragments. Multiple Sequence alignment of skβ‐V1 fragments (hypervariable region1 of the β‐domain)from nucleotide “445 to 655” of sk gene, corresponding to the amino acid residues “147 to 218” of the SK of the strains used in this study (G88, STAB902 and ALAB49; Genbank Accession numbers: HM390000.1, CP007041.1 and AY234134, respectively) together with the well‐known strains that were employed for construction of phylogenetic tree in Fig. 1A (Genbank Accession numbers: NS10; EU352637.1, NS32; EU352630.1, 5448; CP008776.1, NS414; EU352621.1, NS501; EU352616.1, NZ131; CP000829.1). The alignment was created using MEGA software [14]. Conserved (identical) nucleotides are indicated by dots. Fig. S2 . Schematic illustration for construction of the parental SK‐encoding plasmids. For construction of the three parental constructs, the genomic DNA from culture of the three targeted streptococci (G88, STAB902 and ALAB49) grown in BHI (brain heart infusion) broth, were isolated by DNA extraction kit (Qiagen, USA) according to the manufacturer’s protocols. The extracted genomic DNA was used as template for PCR‐based amplification of the coding region of sk genes (lacking the signal peptide sequence) using a pair of primers with inserted restriction sites (NdeI‐ XhoI) for direct cloning into pET26b vector (forward primer; NdeI‐SKf: 5׳‐GACGAGACATATGATTGCTGGACCTGAGTG‐3׳; reverse primer; XhoI‐SKr 5׳‐GACACTCGAGTTTGTCGTTAGGGTTATCAG‐3׳, the sequences corresponding to restriction sites are underlined). The resulting amplified fragments were digested with NdeI and XhoI and cloned into the same sites of pET26b expression vector downstream of T7 promoter, in tandem with the fused C‐terminus 6XHis‐tag to provide the three parental SK‐encoding vectors (pET26b‐SKG88, pET26b‐SKALAB49 and pET26b‐SKSTAB902). All cloning steps were performed according to standard procedures [15]. ATG stands for vector‐derived, translation‐start codon; MCS, multi [file FEB4-9-1259-s001.docx]

**Supplementary Files**

NS10 AAAAACCAAT ACAAACTCCA GCAAAATCTG TTGATATAAG ATATACTGTA CAGTTTACTC [505]

ALAB49 .......... .......... .......... .......... .......... .......... [505]

NS32 .......... .......... .......... .......... ....G..... ........C. [505]

G88 .......... .....AC.A. ..G....... .....G.GGA .......... .......... [505]

STAB902 ........G. .....A..A. ..G....... .....G.G.A .......... .......... [505]

5448 ........G. .....A..A. .......... .....G..GA .......... .......... [505]

NS414 CT...A.CG. T..C.ACT.. ..TG..CGC. ..A.CG.C.A C...GAA..G AGC...GTCT [505]

NS501 CT...G.CG. T..C.ACT.. ..TG..CGC. ..A.CG.C.A C...GAA..G AGC...GTCT [505]

NZ131 CT...G.CG. T..C.ACT.T ..TG..CGC. ..A.CG.C.A C...GAA..G AGC...GTCT [505]

NS10 CTTTAAACCC TGATGATGAT TTCAAACCAG TTCTCAAAGA TACTAAACTA TTGAAAACAT [565]

ALAB49 .C..G..... .......... .......... .......... .......... .......... [565]

NS32 .C........ .......... ....C..... G.A....... .......... .......... [565]

G88 .C........ ......C... ....G..... G......... ......G... .........C [565]

STAB902 .......... ......C... ....G..... G......... ......G... .........C [565]

5448 .......... ......C... ....G..... GG........ ......G... .........C [565]

NS414 .CGA..CAGG A...TTA..C ..T.CG...T CGT.A.G... .CGAT.C.AT ....CC...C [565]

NS501 .CGA..CAGG A...TTA..C ..T.C...GT .GT.A.G.A. CCAAT.C.AT ....CC...C [565]

NZ131 .CGA..CAGG A...TTA..C ..T.C...GT .GT.A.G.A. CCAAT.C.AT ....CC...C [565]

NS10 TAGCTATCGG TGACACCATC ACATCTCAAG AATTACTAGC TCAAGCACAA AGCATTTTAA [625]

ALAB49 .......... C......... .....C.... .......... .......... .......... [625]

NS32 .......... C......... .....C.... .......... .......... .......... [625]

G88 .......... .......... .......... .......... .......... .......... [625]

STAB902 .......... .......... .......... .......... .......... .......... [625]

5448 .......... .......... .......... .......... .......... .......... [625]

NS414 .G..AG.T.. ....T.TC.T T....A.... .G...GC... AATT..C... TTT..CC..T [625]

NS501 .G..AG.T.. ....T.TC.T T....A.... .G...GC... GATT..C... TTT..CC..T [625]

NZ131 .G..AG.T.. ....T.TC.T T....A.... .G...GC... AATT..C... TTT..CC..T [625]

NS10 ACGAAAGCCA TCCAAATTAT ACGATTTATG [655]

ALAB49 .......... ....G..... .......... [655]

NS32 .......... .......... ......C... [655]

G88 ..A...C... C...GGC... .......... [655]

STAB902 ..A...C... C...GGC... .......... [655]

5448 ..A...C... C...GGC... .......... [655]

NS414 CAA..GAG.. ....G..... .TC...ACAA [655]

NS501 CAA...AGT. ....G..... .TC...ACAA [655]

NZ131 CAA...AA.. ....G..... .TC...ACAA [655]

**Supplementary Fig. 1: Multiple DNA Sequence alignment of *skβ-*V1 fragments.** Multiple Sequence alignment of skβ-V1 fragments (hypervariable region1 of the β-domain)from nucleotide “445 to 655” of *sk* gene, corresponding to the amino acid residues “147 to 218” of the SK of the strains used in this study (G88, STAB902 and ALAB49; Genbank Accession numbers: HM390000.1, CP007041.1 and AY234134, respectively) together with the well-known strains that were employed for construction of phylogenetic tree in Fig. 1A (Genbank Accession numbers: NS10; EU352637.1, NS32; EU352630.1, 5448; CP008776.1, NS414; EU352621.1, NS501; EU352616.1, NZ131; CP000829.1). The alignment was created using MEGA software [14]. Conserved (identical) nucleotides are indicated by dots.


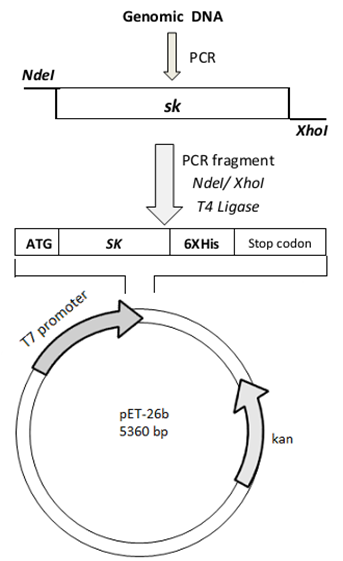


**Supplementary Fig. 2:** **Schematic illustration for construction of the parental SK-encoding plasmids.** For construction of the three parental constructs, the genomic DNA from culture of the three targeted streptococci (G88, STAB902 and ALAB49) grown in BHI (brain heart infusion) broth, were isolated by DNA extraction kit (Qiagen, USA) according to the manufacturer’s protocols. The extracted genomic DNA was used as template for PCR-based amplification of the coding region of *sk* genes (lacking the signal peptide sequence) using a pair of primers with inserted restriction sites (*Nde*I- *Xho*I) for direct cloning into pET26b vector (forward primer; *Nde*I-SKf: 5׳-GACGAGACATATGATTGCTGGACCTGAGTG-3׳; reverse primer; *Xho*I-SKr 5׳-GACACTCGAGTTTGTCGTTAGGGTTATCAG-3׳, the sequences corresponding to restriction sites are underlined). The resulting amplified fragments were digested with *Nde*I and *Xho*I and cloned into the same sites of pET26b expression vector downstream of T7 promoter, in tandem with the fused C-terminus 6XHis-tag to provide the three parental SK-encoding vectors (pET26b-SK_G88_, pET26b-SK_ALAB49_ and pET26b-SK_STAB902_). All cloning steps were performed according to standard procedures [15]. ATG stands for vector-derived, translation-start codon; MCS, multiple cloning sites; 6His-tag is the tag derived from the vector.


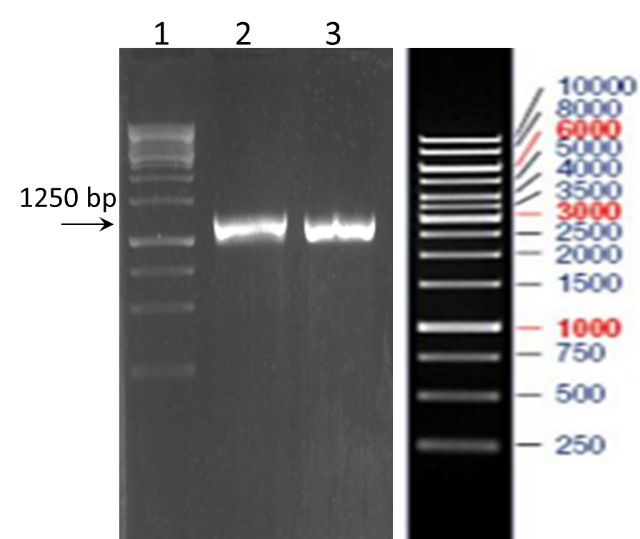


**Supplementary Fig. 3: Analysis of the PCR-amplified *sk* genes by agarose gel (1%) electrophoresis**. The coding region of *sk* gene (lacking the signal peptide sequence) was amplified by PCR using SKf and SKr primers. PCR reactions resulted in a single band of the expected length (1250bp) of *sk* genes. Lane1: DNA Marker 1kb (Thermo scientific SM0311), Lane2 and 3: PCR products of *skg88* and *skstab902* gene from genomic DNA. The corresponding bands were indicated by arrows. The sizes of the bands of DNA marker are illustrated on the right.


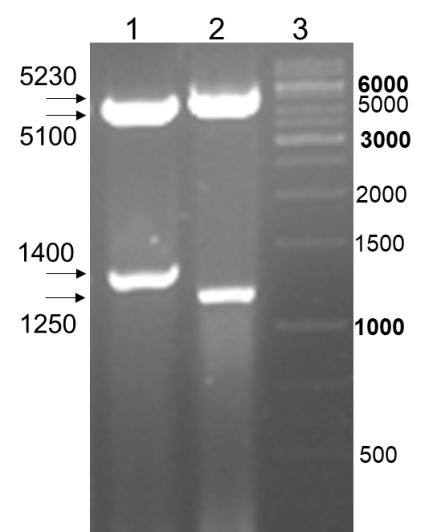


**Supplementary Fig. 4**: **Agarose gel (1%) electrophoresis of the restriction enzyme analysis of the recombinant vector pET26b-SKG_88._** Lane 1: Digested pET26b-SKG_88_ by *Bst*EII, produced two bands with the approximate size of 1400 and 5100 bp. Lane 2: Digested pET26b-SKG_88_ by *Nde*I*-Xho*I, yielded 5230 and 1250 bp fragments corresponding to vector and PCR fragments, respectively. The corresponding bands were indicated by arrows. Lane 3: DNA Marker 1kb (Thermo scientific SM0311). The same analysis for the other two parental constructs pET26b-SK_ALAB49_ and pET26b-SK_STAB902_ produced the same results (not shown).

A

B


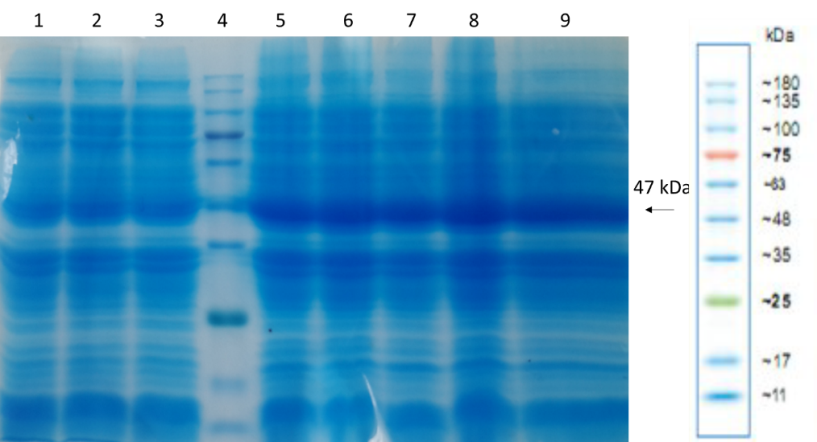
**
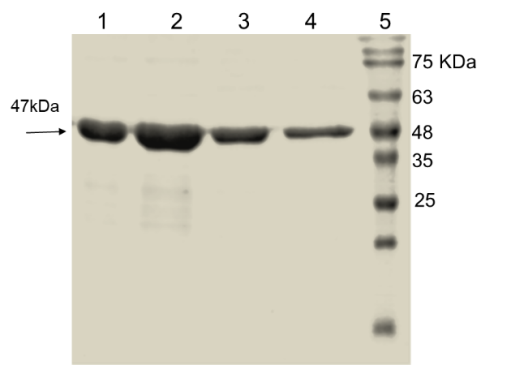
**

**Supplementary Fig. 5**: **Analysis of the protein expression and purification by SDS-PAGE (12%).** For protein expression, first the transformation of *E. coli* Rosetta cells (Novagen, USA) with the seven SK-encoding recombinant vectors (three parental vectors: pET26b-SK_G88_, pET26b-SK_ALAB49_ and pET26b-SK_STAB902_ and four domain-exchanged chimeric vectors: pET26b-SK_C1_ to pET26b-SK_C4_) by standard CaCl_2_ method was performed. Subsequently, protein expression was induced at OD_600_ of 0.5–0.6 by isopropyl-β-D-thio-galactoside (IPTG) to a final concentration of 1 mM for 3 hours at 37°C. Finally, cells were harvested by centrifugation and stored at -20°C for purification steps. Purification of His-tagged SK proteins from induced *E.coli Rosetta* cells was performed under native conditions, using nickel-nitriloacetic acid (Ni-NTA) affinity chromatography and according to manufacturer’s protocol (QIAexpressionist, 2002, Qiagen company website). Briefly, the cell pellets were resuspended in binding buffer (50 mM NaH_2_PO_4_, 300 mM NaCl, 10 mM imidazole) with 0.5 mg/ml lysozyme at 2–5 ml per gram wet weight. After incubation on ice for 30 min, the cells were disrupted by sonication, and supernatant was collected after centrifugation at 10,000 g for 20-30 min at 4°C. After addition of 1ml resin Ni-NTA to the clear lysate, the mixture was shaken at 4°C for 60 minutes, loaded on column and washed 4 times with 4 ml wash buffer (50 mM NaH_2_PO_4_, 300 mM NaCl, 20 mM imidazole) then 4 times with 0.5 ml elution buffer (50 mM NaH_2_PO_4_, 300 mM NaCl, 250 mM imidazole) (QIAexpressionist 2002). SDS-PAGE analyses were performed according to standard procedures [15]. **(A)** Analysis of the bacterial crude extracts for expression by pET26b-SK_G88_. Lanes 1-3; un-induced bacterial cells, Lane 4; protein marker 10-180 kDa (SM7012 CinnaGen, Iran), Lanes 5-8; corresponds to IPTG induced cells with 0.3, 0.5, 0.7, 1 and 1.2 mM IPTG, respectively. **(B)** Analysis of protein purification steps for pET26b-SK_G88_. Lanes 1- 4 represent the elution E1 to E4, respectively collected after protein extraction under native condition. Lane5: protein marker 10-180 kDa (SM7012 CinnaGen). Arrows indicate the location of 47 kDa (SK). Analysis of other constructs for expression and purification produced the same results (not shown).

**
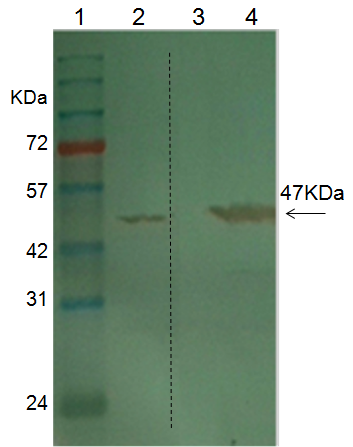
**

**Supplementary Fig. 6: Confirmation of the expressed proteins by western blotting.** Western blotting was performed according to the standard protocols [15]. Briefly, proteins were transferred from SDS-PAG to the nitrocellulose membrane and the membrane was blocked by 5% BSA. Mouse anti-His monoclonal antibody (Qiagen, USA) was used as the primary antibody and goat anti-mouse IgG conjugated to HRP (Horse Radish peroxidase) (Qiagen, USA) as the secondary (tracking) antibody. Detection of the bands was by 3, 3- diaminobenzidine (DAB) (Qiagen, USA). Western-blot analysis for SK2a_G88_ and SK_C1_ proteins are shown. Lane 1; molecular weight marker (SM7012, Cinnagen, Iran), lanes 2 and 4; crude lysis of *E. coli* Rosetta cells after induction by IPTG (1mM), expressing SK2a_G88_ and SK_C1_, respectively, lane 3; crude lysis of *E. coli* Rosetta cells before induction (no band was observed). Lanes are spliced together to remove an intervening lane and the vertical dotted line is at the location of the spliced lanes. The arrow indicates the location of 47 kDa (SK).


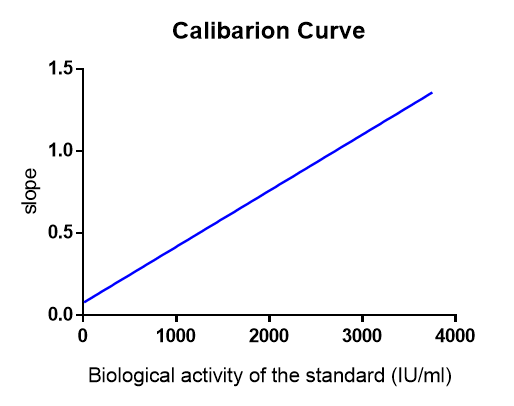


**Supplementary Fig. 7**: **Calibration curve for standard SK.** Serial dilutions of Streptase® (CSL, Behring, Germany), a commercially available standard SK, were used to prepare the standard calibration curve based on Hydrolysis of S-2251 by Pg, as explained in Fig.2 and Fig.3.

SKC9542 IAGPEWLLDR PSVNNSQLVV SVAGTVEGTN QDISLKFFEI DLTSRPAHGG KTEQGLSPKS [ 60]

SKALAB ...Y...... .......... .M..I....D KKVFIN.... ....Q..... .......... [ 60]

SKG88 ---------- -------... .......... .......... ......D... ....N..... [ 60]

SkSTAB ...Y...P.. .P........ S.AG.V...D KKVFIN.... ....QH.... .......... [ 60]

SKC9542 KPFATDSGAM PHKLEKADLL KAIQEQLIAN VHSNDDYFEV IDFASDATIT DRNGKVYFAD [120]

SKALAB ......N... .......... .....R.... .....G.... .......... ..DDNI...N [120]

SKG88 .......... .......... .......... .......... .......... .......... [120]

SKSTAB ......N... .......... ....K..... .....G.... .......... .......... [120]

SKC9542 KDGS**VTLPTQ PVQEFLLSGH VRVRPYKEKP** **IQNQAKSVDV EYTVQFTPLN PDDDFRPGLK** [180]

SKALAB Q......... .I.Q...R.. .......... ..TP.....I R......... .....K.V.. [180]

SKG88 .......... .......K.. .......... .......... .......... .......... [180]

SKSTAB .......... .......... .......... v......... K......... .......... [180]

SKC9542 **DTKLLKTLAI GDTITSQELL** **AQAQSILNKT HPGYTIYERD SSIVTHDNDI FRT**ILPMDQE [240]

SKALAB .......... .......... ........ES ..D....... .......... ......T... [240]

SKG88 .......... .......... .......... .......... .......... .......... [240]

SKSTAB .......... .......... .......... .......... .......... .......... [240]

SKC9542 FTYHVKNREQ AYEINKKSGL NEEINNTDLI SEKYYVLKKG EKPYDPFDRS HLKLFTIKYV [300]

SKALAB .......... ..QNDN.T.. KK.TK..... .....I.... .......... .......... [300]

SKG88 .......... ..R....... .......... .......... .......... .......... [300]

SKSTAB .......... ..G....... .......... .......... .S........ .......... [300]

SKC9542 DVNTNELLKS EQLLTASERN LDFRDLYDPR DKAKLLYNNL DAFGIMDYTL TGKVEDNHDD [360]

SKALAB ..D.KD.... .......... .......... .......... ...D...... .........K [360]

SKG88 .......... .......... .......... ....V..... .......... .......... [360]

SKATAB .......... .......... .........C .......... .......... .........K [360]

SKC9542 TNRIITVYMG KRPEGENASY HLAYDKDRYT EEEREVYSYL RYTGTPIPDN PNDK [414]

SKALAB N..VV..... ...K.AKG.. .......L.. .......... .D........ .... [414]

SKG88 .......... .......... .......... .....A.... ..S....... .... [414]

SKSTAB N...V..... ...K.AKG.. .......L.. .....A.... .D.E...... .... [414]

**Supplementary Fig. 8: Amino acid sequence alignment of SK proteins corresponding to reference strain SK_9542_ *(S. equisimilis*, ATCC9542, the commercial source for production of SK), SK2b_ALAB49_, SK2a_G88_, and SK2a_STAB902_.** The alignment was created using MEGA6 software [14]. Conserved (identical) amino acids in the alignment are indicated by dots. The exchanged fragments are highlighted.
